# Abdominal subcutaneous adipose tissue insulin resistance and lipolysis in patients with non-alcoholic steatohepatitis

**DOI:** 10.1111/dom.12272

**Published:** 2014-03-11

**Authors:** M J Armstrong, J M Hazlehurst, D Hull, K Guo, S Borrows, J Yu, S C Gough, P N Newsome, J W Tomlinson

**Affiliations:** 1 Centre for Liver Research and NIHR Liver Biomedical Research Unit, University of BirminghamBirmingham, UK; 2 Centre for Endocrinology, Diabetes and Metabolism, Institute of Biomedical Research, School of Clinical and Experimental Medicine, University of BirminghamBirmingham, UK; 3 NIHR/Wellcome Trust Clinical Research Facility, Queen Elizabeth HospitalBirmingham, UK; 4 School of Sport, Exercise & Rehabilitation Sciences, University of BirminghamBirmingham, UK; 5 Oxford Centre for Diabetes, Endocrinology and Metabolism, and NIHR Oxford Biomedical Research CentreOxford, UK

**Keywords:** adipose tissue, fatty liver, insulin sensitivity, lipolysis, steatohepatitis

## Abstract

**Background:**

Systemic insulin resistance (IR) is a primary feature in non-alcoholic steatohepatitis (NASH), however, there remain limited data on tissue-specific insulin sensitivity *in vivo*.

**Methods:**

We examined tissue-specific (adipose, muscle and liver) insulin sensitivity and inflammation in 16 European Caucasian patients with biopsy-confirmed NASH and in 15 healthy controls. All underwent a two-step hyperinsulinaemic euglycaemic clamp incorporating stable isotope measurements of carbohydrate and lipid metabolism with concomitant subcutaneous adipose tissue (SAT) microdialysis.

**Results:**

Hepatic and muscle insulin sensitivity were decreased in patients with NASH compared with controls, as demonstrated by reduced suppression of hepatic glucose production and glucose disposal (Gd) rates following insulin infusion. In addition, rates of lipolysis were higher in NASH patients with impaired insulin-mediated suppression of free fatty acid levels. At a tissue specific level, abdominal SAT in patients with NASH was severely insulin resistant, requiring >sixfold more insulin to cause ½-maximal suppression of glycerol release when compared with healthy controls. Furthermore, patients with NASH had significantly higher circulating levels of pro-inflammatory adipocytokines than controls.

**Conclusion:**

NASH patients have profound IR in the liver, muscle and in particular adipose tissues. This study represents the first *in vivo* description of dysfunctional SAT in patients with NASH.

## Introduction

Non-alcoholic fatty liver disease (NAFLD) is reaching epidemic proportions, affecting up to 30% of the general population and 70–90% of individuals with type 2 diabetes and/or obesity 1, and is now expected to become the leading indication for liver transplantation by 2020 2. Furthermore, NAFLD and in particular the inflammatory (with or without fibrosis) component of non-alcoholic steatohepatitis (NASH), is associated with a significant risk of developing type 2 diabetes, chronic kidney disease, and cardiovascular morbidity and death 1,3. A better understanding of the key components of the pathogenesis of NASH is therefore needed to provide new therapeutic approaches and thus prevent progressive liver disease and extra-hepatic complications.

Systemic insulin resistance (IR) is recognized as one of the main pathogenic factors in NASH 4,5. Using hyperinsulinaemic euglycaemic clamp techniques (coupled with stable isotopes), several studies have identified the liver (with increased glucose production) and muscle [with decreased glucose disposal (Gd)] as the key sites of increased IR in patients with NASH 5–9. Recent studies have recognized the importance of adipose tissue, as the principal source of fatty acids (≈60%) for the liver, in driving lipid synthesis in both healthy individuals 10 and NASH patients 11. Adipose tissue is a highly insulin responsive tissue. In an insulin-sensitive state, insulin promotes lipid storage [through fatty acid uptake, re-esterification and *de novo* lipogenesis (DNL)] and inhibits triglyceride lipolysis, the process whereby triglycerides are hydrolysed to release non-esterified fatty acids (NEFAs) from their glycerol backbone. Studies in patients with NASH have inferred changes in adipose tissue insulin sensitivity through systemic measures of circulating NEFA which are elevated in both the fasting state and under hyperinsulinaemic conditions 6–8,12. Importantly, this appears to be independent of the degree of obesity 13.

Adipose tissue dysfunction is considered to be a major contributory factor of NASH, by means of the resultant ‘lipotoxicity’ inducing both hepatic IR and skeletal muscle IR 14. Studies that have been published to date have, however, solely focused on quantifying whole-body lipolysis using either circulating NEFA [i.e. quantified by adipose insulin resistance (ADIPO-IR) index = fasting NEFA × insulin] 8,9,12 or the rate of systemic appearance of labelled glycerol/palmitate isotopes 6,13,15. In particular, no studies have assessed the response of local adipose tissue to the action of insulin, which provides greater insights into the functional relevance of adipose tissue. A greater understanding of which adipose depots are dysfunctional in NASH patients would greatly enhance our knowledge in developing new targeted therapies. Traditionally, visceral adipose tissue (VAT) has been recognized as a major contributor to IR and metabolic conditions including NASH, due to its close proximity to the portal vein and abundance of pro-inflammatory mediators 16,17. However, as VAT only contributes to 15–20% of circulating NEFA pool 18,19, researchers have questioned whether overspill from abdominal subcutaneous adipose tissue (SAT) plays a more significant role. Indeed, while several studies have linked abdominal SAT with indices of IR in subjects with and without metabolic syndrome 20–23, none have examined it in relation to NASH.

Adopting an integrative physiological approach with functional measures of lipid and carbohydrate flux, we have performed a clinical study to determine the relative contribution of tissue-specific insulin sensitivity, notably in SAT, in patients with biopsy-proven NASH in comparison with a healthy control cohort.

## Research Design and Materials

The clinical protocols received full ethical approval from Leicestershire, Northamptonshire and Rutland (ref. 10/H0402/32) and South Birmingham (ref. 10/H1207/15) Local Research Ethics Committees. All adult subjects gave informed written consent prior to participation.

### Study Subjects

#### NASH Patients

Sixteen patients with a definitive diagnosis of NASH on liver biopsy within 6 months of the study were recruited. The histological diagnosis was made using well-established criteria 24 by two independent liver histopathologists. The subjects were of adult age (18–70 years) and had a body mass index (BMI) ≥ 25 kg/m^2^. Patients with co-existing type 2 diabetes were diet-controlled or were on a stable dose of metformin ± gliclazide for a minimum of 3 months prior to the study and had a glycated haemoglobin 1c (HbA1c) < 9.0%. Participants were excluded if they had a history of excess alcohol consumption (females >14 units/week and males >21 units/week), liver disease of other aetiology, decompensated cirrhosis (Child's Pugh B or C), recent or concomitant drug use of inducers of hepatic steatosis/weight-inducing therapy, and significant active medical illnesses (with the exception of type 2 diabetes). All patients with no previous diagnosis of type 2 diabetes underwent a 75 g oral glucose tolerance test (OGTT).

#### Healthy Volunteers

Fifteen healthy volunteers (9 males : 6 females; mean age 33 ± 2 years) were recruited by use of a local advertisement. All controls were asymptomatic, non-diabetic, were taking no regular medication and had no significant medical history of note. Female controls had pregnancy excluded and were not taking any form of hormonal contraception. In the healthy control cohort, all consumed alcohol within recommended limits, had normal liver function tests (LFTs) and had normal levels of non-invasive markers of hepatic injury (serum cytokeratin-18) and fibrosis [serum enhanced liver test, enhanced liver fibrosis (ELF)]. Furthermore, all controls had a negative NAFLD liver fat score (<−0.640) and estimated liver fat <3.0% based on the Kotronen et al. equations, which were originally validated with ^1^H-magnetic resonance spectroscopy (MRS) 25; 5 of 15 subjects underwent a hepatic MRS, as part of a separate study, and in keeping with the Kotronen equations, had hepatic steatosis excluded (<2.5%).

### Study Design

All participants underwent a two-step hyperinsulinaemic euglycaemic clamp incorporating stable isotopes with concomitant SAT microdialysis at the NIHR/Wellcome Trust Clinical Research Facility (WTCRF, Birmingham, UK) (Figure S1, Supporting information).

#### Hepatic DNL

At 17:00 hours, participants were admitted to the WTCRF and total body water was estimated by bioimpedance (Tanita BC418MA, Amsterdam, the Netherlands). A standardized meal (carbohydrate 45 g, protein 23 g and fat 20 g) was provided at 17:00 hours, after which participants remained fasted until the end of the clamp at 14:00 hours the next day. To determine rates of DNL, participants were given oral deuterated water, ^2^H_2_O (3 g/kg total body water in two divided doses), at 18:00 and 22:00 hours followed by *ad libitum* drinking water enriched with 0.4% ^2^H_2_o.

#### Two-Step Hyperinsulinaemic Euglycaemic Clamp

At 08:00  hours the next morning fasting blood samples were taken by standard needle venepuncture prior to starting the two-step hyperinsulinaemic euglycaemic clamp. Arterialized blood was sampled to determine the blood glucose concentration at which to maintain (‘clamp’) the participant throughout the study using an YSI 2700 machine (YSI life sciences, Fleet, Hampshire, UK). An intravenous bolus of U-[^13^C]-glucose (2 mg/kg body weight; CK gas limited, Hook, UK) was administered over 1 min followed by a constant infusion rate (0.02 mg/kg/min) for 6 h until the end of the clamp. Steady state blood samples were taken at three time points during the final 30 min of the 2-h basal phase. At 10:00 hours, low-dose insulin (Actrapid; Novo Nordisk, Copenhagen, Denmark) was infused at 20 mU/m^2^/min. At 10:04 hours a concomitant infusion of 20% glucose enriched with U-[^13^C]-glucose to 4% was commenced. Arterialized blood samples were taken at 5 min intervals and the 20% glucose infusion rate was changed to maintain fasting glycaemic levels. Steady state blood samples were taken at three time points in the final 30 min of the 2-h low-dose insulin infusion. The insulin infusion rate was then increased to 100 mU/m^2^/min (high-dose) for 2 h with sampling as described above. Rates of hepatic endogenous glucose production (EGP) and Gd were calculated by using modified versions of the Steele Equations 26,27.

#### Adipose Microdialysis

A microdialysis catheter (CMA microdialysis AB, Solna, Sweden) was inserted after local anaesthetic (5 ml 1% lignocaine) was injected into the abdominal SAT (minimum depth 1 cm), 10 cm lateral to the umbilicus, prior to commencing the clamp. Thereafter, micro-dialysate samples were collected into micro-vials (0.3 µl/min) every 30 min until the end of the clamp.

### Data Collection and Analysis

#### Clinical and Biochemical Parameters

Participant demographics and clinical/biochemical measures were recorded at the study visit. These included 75 g OGTT, anthropometry (including bioimpedance), fasting haematological/biochemical bloods and non-invasive serum markers of liver injury [cytokeratin-18 (CK-18), ELF test, Kotronen score)]. Serum insulin, NEFA and adipocytokines [adiponectin, leptin, resistin, tumour necrosis factor alpha (TNF-α), high sensitivity C-reactive protein (hs-CRP), interleukin-6 (IL-6), IL-17, chemokine ligand-2 (CCL-2), CCL-3, CCL-4 and CCL-5] were measured using commercially available kits. Detailed descriptions of the above are available in Appendix S1.

#### Abdominal SAT Microdialysis

Microdialysate samples were analysed using a mobile photometric, enzyme-kinetic analyser (CMA Iscus Flex) for glycerol concentration. The rate of interstitial glycerol release represented the magnitude of SAT lipolysis in the fasted state and in response to insulin.

#### Stable Isotope Mass Spectrometry Analysis

The enrichment of U-[^13^C]-glucose in plasma was determined by gas chromatography–mass spectrometry (model 5973; Agilent technologies, Cheshire, UK). Deuterium enrichment of the body water pool was measured using the Gasbench II (http://www.thermo.com), an automated H_2_/H_2_O equilibration device, coupled on-line to a ThermoFinnigan Deltaplus XP Isotope Ratio Mass Spectrometer (IRMS; ThermoFinnigan MAT GmbH, Bremen, Germany). The full methods have been previously described in detail 28. Deuterium enrichment in the palmitate fraction of total plasma triglycerides was measured on an automated GC/TC/IRMS system (ThermoFinnigan Delta Pus XP; http://www.thermo.com).

#### Contribution of Hepatic DNL to Total Palmitate Synthesis

The percentage contribution of hepatic DNL to endogenous palmitate synthesis was determined by the incorporation of ^2^H_2_O in the palmitate present in the plasma total triglyceride pool, as previously described 28. This percentage was calculated from the increase in the ^2^H/^1^H ratio in the palmitate methylester of the total triglyceride fraction and in the water of plasma samples taken before (17:00 hours, at admission) and 14 h after the initial ingestion of the ^2^H_2_O tracer (08:00 hours, before the start of the hyperinsulinaemic euglycaemic clamp). The following formula was used: % hepatic DNL contributes to endogenous palmitate synthesis = (delta ^2^H/^1^H ratio in palmitate methylester)/(delta ^2^H/^1^H ratio in waterpool) × (34/22) × 100%. In the equation, 34 is the total number of H-atoms in palmitate methylester and 22 is the number of water molecules incorporated into palmitate via DNL as observed in previous rodent studies 29 and currently used in human studies 30.

### Statistical Analysis

Descriptive statistics was applied to characterize the NASH and healthy volunteer cohorts. Continuous clinical and laboratory variables are reported as means and standard error (s.e.) as all variables had parametric distribution on D'Agostino and Pearson Omnibus Normality testing. Categorical variables are reported as number and percentages. Area under the curve (AUC) analysis was performed using the trapezoidal method for interstitial glycerol release during the clamp. For comparison of single variables, unpaired Student *t*-tests were used (or non-parametric equivalents where data were not normally distributed). Where repeated samples were taken repeated-measures one-way analysis of variance (anova) was used, incorporating the Dunnett's test for multiple comparisons. The significance level was set at p < 0.05. All analyses were performed using the graphpad prism 5.0 software package.

## Results

### Participant Characteristics

Participant demographics and clinical characteristics are summarized in Table1. NASH subjects were significantly older (54.4 ± 2.1 vs. 33.1 ± 2.2 years; p < 0.0001) and had a higher BMI (34.3 ± 1.0 vs. 26.7 ± 1.0 kg/m^2^; p < 0.0001) and abdominal fat mass on bioimpedance (20.3 ± 1.5 vs. 12.0 ± 1.5 kg; p = 0.0011). Of the 16 subjects with NASH, 5 had mild-moderate fibrosis (Kleiner F1-F2) and 9 had advanced fibrosis (F3-F4). NASH subjects had significantly higher serum levels of liver enzymes [alanine transaminase (ALT) 68.7 ± 11 vs. 18.9 ± 2.6 IU/l; p = 0.0001], serum CK-18 M30 levels (544 ± 116 vs. 161 ± 9.8 IU/l; p = 0.0034) and ELF test (9.20 ± 0.3 vs. 7.34 ± 0.1; p < 0.001); values for all these parameters were within accepted reference ranges in the healthy volunteers.

**Table:** Demographics and clinical parameters of 16 patients with NASH and 15 healthy controls. Values are mean (s.e.), unless stated. All blood parameters were fasting samples. Comparisons of continuous variables were made with unpaired Student's *t*-test, and categorical variables with fisher exact/chi-squared test

	NASH (n = 16)	Controls (n = 15)	p-Value
Demographics			
Male sex, n (%)	11 (68.8)	9 (60.0)	0.716
Age (years)	54.4 (2.1)	33.1 (2.2)	**<0.0001**
Ethnicity, n (%)			
Caucasian	16 (100)	14 (93.3)	0.484
Asian	0 (0)	1 (6.7)	
Metabolic parameters			
Type 2 diabetes, n (%)	7 (43.8)	0 (0)	**0.001**
Impaired glucose tolerance, n (%)	3 (18.8)	0 (0)	
Normal glucose tolerance, n (%)	6 (37.5)	15 (100)	
Fasting glucose (mmol/l)	5.34 (0.24)	4.37 (0.067)	**0.0008**
Fasting insulin (pmol/l)	125.8 (20.8)	43.3 (7.41)	**0.0003**
HbA1c (%)	5.99 (0.21)	—	—
Pre-study OAD treatment, n (%)	8 (50.0)	0 (0)	**0.0024**
Pre-study statin treatment, n (%)	7 (43.8)	0 (0)	**0.0068**
Pre-study anti-hypertensive treatment, n (%)	6 (37.5)	0 (0)	**0.0177**
BMI (kg/m^2^)	34.3 (1.04)	26.7 (0.95)	**<0.0001**
Weight (kg)	100.3 (3.83)	78.5 (3.67)	**0.0003**
Total fat mass (kg)	35.8 (2.64)	20.2 (1.79)	**<0.0001**
Truncal fat mass (kg)	20.3 (1.45)	12.0 (1.51)	**0.0011**
Systolic BP (mmHg)	129.4 (3.43)	129.3 (2.74)	0.982
Waist circumference (cm)	114.1 (2.87)	85.9 (3.07)	**<0.0001**
Total cholesterol (mmol/l)	4.51 (0.20)	4.59 (0.30)	0.891
HDL (mmol/l)	1.11 (0.064)	1.26 (0.11)	0.256
LDL (mmol/l)	3.01 (0.21)	2.73 (0.43)	0.550
Triglycerides (mmol/l)	1.95 (0.26)	1.62 (0.34)	0.438
TSH (µU/l)	2.76 (0.38)	2.01 (0.31)	0.165
Creatinine (µmol/l)	71.3 (3.46)	72.6 (3.47)	0.800
Liver parameters			
AST (IU/l)	55.1 (5.66)	20.4 (1.55)	**<0.0001**
ALT (IU/l)	68.7 (10.6)	18.9 (2.57)	**0.0001**
Alk Phos (IU/l)	155.7 (25.3)	128 (9.64)	0.327
Bilirubin (µmol/l)	13.4 (1.76)	12.0 (1.02)	0.494
Albumin (g/l)	47.1 (0.70)	41.6 (0.77)	**<0.001**
Platelets (×10^9^ l^−1^)	203.3 (14.7)	216.1 (10.5)	0.489
CK-18 M30 (IU/l)	543.4 (115.8)	160.9 (9.83)	**0.0034**
ELF test	9.20 (0.30)	7.34 (0.12)	**<0.0001**
Histology parameters (NASH only)			
Median Kleiner fibrosis score* (IQR)	3.0 (1.0–3.75)	—	—
Median NAFLD activity score† (IQR)	4.5 (4.0–5.0)	—	—

Bold are the p-values that are significant at <0.05. ALT, alanine transaminase; AST, aspartate transaminase; Alk phos, alkaline phosphatase; BMI, body mass index; BP, blood pressure; CK-18, cytokeratin-18; ELF, enhanced liver fibrosis; HbA1c, glycated haemoglobin 1c; HDL, high-density lipoprotein; IQR, interquartile range; LDL, low-density lipoprotein; NAFLD, Non-alcoholic fatty liver disease; NASH, non-alcoholic steatohepatitis; OAD, oral anti-diabetic drug; TSH, thyroid stimulating hormone.

& The Kleiner fibrosis score ranges from 0 to 4, whereby 0 = no fibrosis and 4 = cirrhosis.

† The NAFLD activity score (scored out of 8) is a sum of steatosis (0–3), hepatocyte ballooning (0–2) and lobular inflammation (0–3).

### Systemic IR

**Figure 1:**
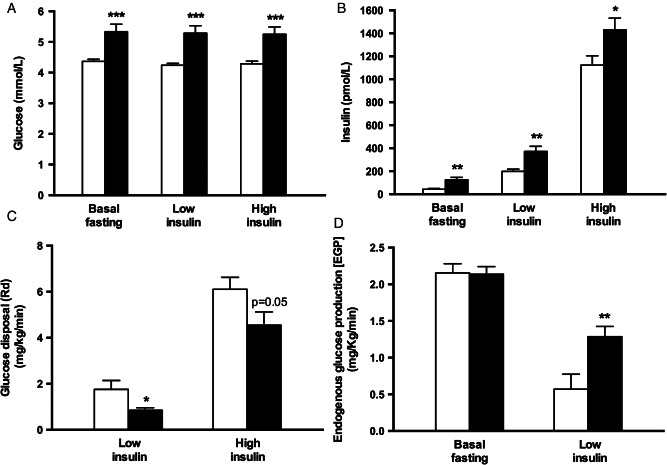
Subjects with non-alcoholic steatohepatitis (NASH) have significant systemic, muscle and hepatic insulin resistance (IR). Circulating glucose (A) and insulin (B) concentrations during the two-step hyperinsulinaemic euglycaemic clamp. The degree muscle and hepatic insulin sensitivity was determined by glucose disposal (C) and suppression of hepatic glucose production (D), respectively. White bar = controls, black bar = NASH. ^&^p < 0.05, ^&&^p < 0.01, ^&&&^p < 0.001 versus controls.

Fasting serum glucose, insulin and homeostatic model assessment insulin resistance (HOMA-IR, 4.40 ± 0.8 vs. 1.19 ± 0.2) were significantly higher in patients with NASH (all p < 0.001) (Figure 1A, B). During the two-step hyperinsulinaemic clamp, NASH subjects had significantly lower weight-adjusted glucose infusion rates in response to low-dose (1.47 ± 0.08 vs. 3.08 ± 0.4 mg/kg/min; p = 0.0008) and high-dose insulin (5.80 ± 0.4 vs. 9.14 ± 0.5 mg/kg/min; p < 0.0001). In keeping with peripheral (largely muscle) IR, weight-adjusted Gd rates were significantly lower in NASH subjects at low-dose (0.85 ± 0.1 vs. 1.76 ± 0.4 mg/kg/min; p < 0.05) and high-dose insulin infusions (4.55 ± 0.6 vs. 6.10 ± 0.5 mg/kg/min; p = 0.05) (Figure 1C).

### Hepatic IR

Although fasting EGP rates were similar in patients with NASH and healthy controls (2.14 ± 0.1 vs. 2.15 ± 0.1 mg/kg/min; p > 0.9; Figure 1D), this was in the context of fasting hyperinsulinaemia (Figure 1B, Table1); changes were consistent with hepatic IR. The hepatic IR index (= EGP × fasting insulin 31) was significantly higher in NASH patients (278 ± 52.7 vs. 90.0 ± 14.9 mg/kg/min pmol/ml; p = 0.0024). In addition, low-dose insulin-mediated suppression of EGP was decreased in patients with NASH (Figure 1D), consistent with hepatic IR (% EGP suppression: 41.0 ± 4.3 vs. 70.2 ± 9.5%; p = 0.008). These differences persisted even after removing patients with type 2 diabetes (n = 6) from the NASH cohort (42.2 ± 5.6 vs. 70.2 ± 9.5%; p < 0.05).

### Hepatic DNL

The percentage contribution of DNL to total endogenous palmitate synthesis was variable across all individuals and although higher in NASH subjects compared with controls [median 4.90 (IQR 3.9–5.6) vs. 2.79 (1.2–6.4); p = 0.16] this did not reach significance.

### Depot-specific adipose tissue IR

**Figure 2:**
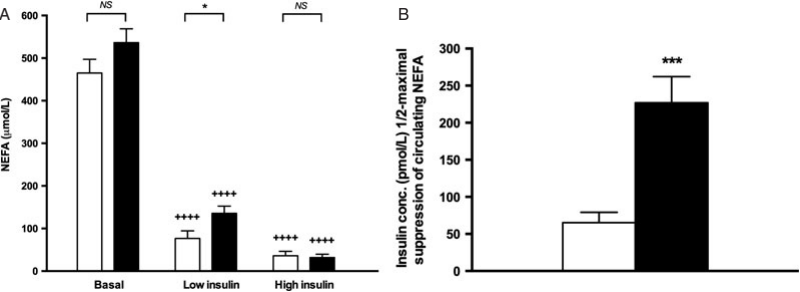
Subjects with non-alcoholic steatohepatitis (NASH) have significant global adipose tissue insulin resistance (IR). (A) Circulating non-esterified fatty acid (NEFA) concentrations at basal and hyperinsulinaemic phases of euglycaemic clamp. (B) As a marker of global adipose tissue insulin resistance, the concentration of circulating insulin concentrations (pmol/l) causing 1/2-maximal suppression of circulating NEFA was calculated. White bar = controls, black bar = NASH. ^&^p < 0.05, ^&&&^p < 0.001 versus controls. ^++++^p < 0.0001 versus basal phase. NS, non-significant.

Circulating NEFA levels were not different between patients with NASH and healthy controls (563 ± 33 vs. 465 ± 32 µmol/l; p = 0.13; Figure 2A). However, taking into account fasting hyperinsulinaemia in patients with NASH, the calculated adipose IR index (fasting NEFA × fasting insulin 31) was significantly elevated (64.4 ± 9.1 vs. 20.5 ± 3.9 mmol/l pmol/l; p = 0.0002) in patients with NASH. Insulin infusion significantly suppressed circulating NEFAs in both NASH and control subjects (p < 0.0001 vs. basal NEFA in each group; Figure 2A). In order to determine insulin sensitivity, using regression analysis, the insulin concentrations causing half-maximal suppression of serum NEFA (INS-½-max NEFA) were calculated for each subject (Figure 2B). INS-½-max NEFA was greater than threefold higher in NASH subjects compared with the controls (227 ± 35 vs. 65.2 ± 14 pmol/l; p = 0.0003) consistent with adipose tissue IR. The significant difference in INS-½-max NEFA remained (195 ± 30 vs. 65.2 pmol/l; p = 0.0002) after removing patients with type 2 diabetes (n = 7) from the analysis.

**Figure 3:**
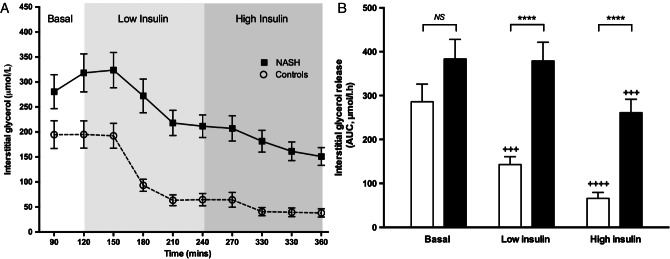
Non-alcoholic steatohepatitis (NASH) is associated with significant abdominal SAT IR. (A) SAT interstitial fluid concentrations of glycerol during the 2-step hyperinsulinaemic euglycaemic clamp. (B) To determine the rate of lipolysis in subcutaneous adipose tissue (SAT) under basal and hyperinsulinaemic conditions area under the curve (AUC) analysis was performed using the trapezoidal method for interstitial glycerol release. Broken line/white bar = controls, solid line/black bar = NASH. ^&&&&^p < 0.0001 versus controls; ^+++^p < 0.001, ^++++^p < 0.0001 versus basal phase. NS, non-significant.

**Figure 4:**
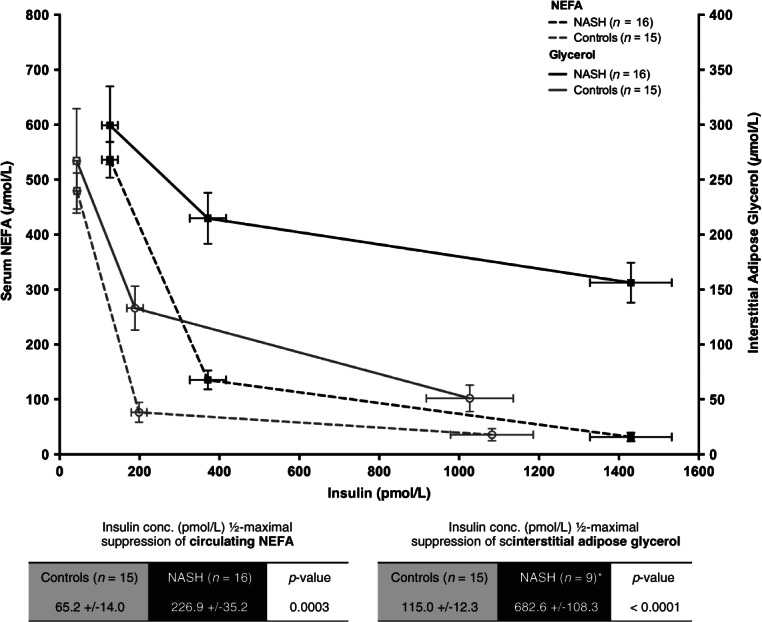
Subjects with non-alcoholic steatohepatitis (NASH) have a disproportionate higher degree of insulin resistance (IR) in subcutaneous adipose tissue (SAT) (sixfold vs. controls) compared with whole-body adipose tissue (threefold vs. controls). Line graph representing the concentrations of circulating non-esterified fatty acid (NEFA) (whole-body lipolysis) and interstitial fluid glycerol (SAT-specific lipolysis) in basal, low-dose and high-dose insulin phases of the euglycaemic clamp. Black lines = NASH (mean ± s.e.), Grey line = control. Sold line = glycerol levels, broken line = NEFA levels.

Interstitial glycerol release assessed using microdialysis was used as a direct measure of abdominal SAT function (Figure 3A, B). In the fasting state, the rate of interstitial glycerol release was not different in NASH subjects compared with controls (383 ± 44 vs. 286 ± 40 µmol/l·h; p = 0.12). In healthy controls, low-dose insulin infusion (20 mU/m^2^/min) significantly suppressed the rate of interstitial glycerol release (Basal: 286 ± 40 vs. low-dose insulin: 143 ± 18 µmol/l·h; p < 0.001), whereas it did not suppress release in NASH subjects (Basal: 383 ± 44 vs. low-dose insulin: 379 ± 43 µmol/l·h; p > 0.05). High dose insulin (100 mU/m^2^/min) suppressed glycerol release in both patients with NASH and in controls, however, the rate of glycerol release remained significantly higher in the NASH subjects compared with controls (261 ± 31 vs. 65.8 ± 14 µmol/l·h; p < 0.0001; Figure 3B). Furthermore, the INS-½-max glycerol was sixfold higher in the NASH subjects compared with controls (p < 0.0001; Figure 4, summary box). All of the above comparisons remained significant after excluding subjects with type 2 diabetes (n = 7) from the NASH cohort (Figure S2).

### Serum Adipocytokines and Inflammatory Cytokines

**Figure 5:**
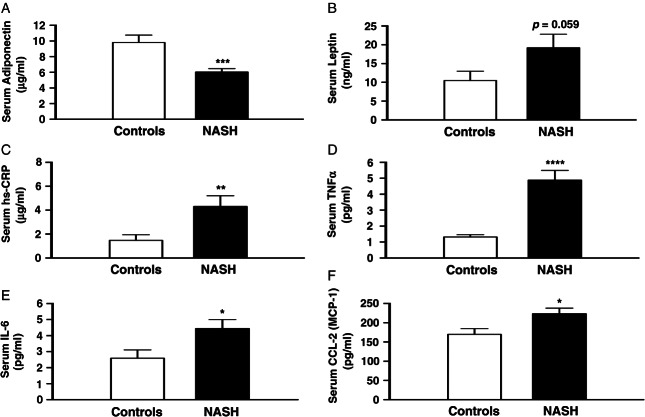
Subjects with non-alcoholic steatohepatitis (NASH) have significantly lower levels of fasting adiponectin (A) and higher levels of fasting pro-inflammatory adipocytkines [(B) leptin, (C) high sensitivity C-reactive protein (hs-CRP), (D) tumour necrosis factor alpha (TNF-α), (E) interleukin-6 (IL-6) and (F) chemokine ligand-2 (CCL-2)/MCP-1]. ^&^p < 0.05, ^&&^p < 0.01, ^&&&^p < 0.001, ^&&&&^p < 0.0001 versus controls.

Subjects with NASH had significantly higher fasting circulating levels of TNF-α (p < 0.0001), hs-CRP (p < 0.05), IL-6 (p < 0.05) and CCL-2 (p < 0.05) than controls (Figure 5). Serum adiponectin levels (p = 0.001) were significantly lower in NASH subjects, with a non-significant trend towards higher circulating leptin compared with controls (p = 0.059). The resultant leptin : adiponectin ratio was 2.5-fold higher in NASH subjects than controls (3.22 ± 0.5 vs. 1.27 ± 0.4; p = 0.0032). There were no significant differences in IL-17, resistin and chemotactic cytokines CCL-3, CCL-4 and CCL-5 (RANTES). With the exception of CCL-2 (p = 0.09), differences in TNF-α (p < 0.0001), hs-CRP (p < 0.05), IL-6 (p < 0.05) and adiponectin remained significant after excluding subjects with type 2 diabetes (n = 7) from the NASH cohort (Figure S3).

## Discussion

The data from this study have begun to address the tissue-specific contributions make to global IR seen in patients with NASH. Using novel techniques that have functional readouts of insulin-regulated processes in a tissue-specific manner allows an assessment of the contribution of the liver (EGP and DNL), skeletal muscle (Gd), and adipose tissue (circulating NEFA and adipose microdialysis) to systemic IR. By doing so, we have not only demonstrated significant IR at the level of the liver, muscle and adipose tissue, but also by measuring depot-specific glycerol release, our study represents the first *in vivo* description of dysfunctional abdominal SAT in patients with NASH.

We observed significant levels of hepatic and muscle IR in NASH subjects, as represented by impaired insulin-mediated suppression of hepatic glucose production and stimulated muscle Gd (weight-adjusted), respectively. In keeping with previous studies 6,13,32, the level of hepatic and muscle IR remained significant when patients with type 2 diabetes were removed from the analysis. Notably, we only saw a non-significant trend towards higher levels of fasting DNL in NASH subjects compared with healthy controls (4.9 vs. 2.8%; p = 0.16). Even though the low levels of fasting DNL in healthy subjects were consistent with the literature (i.e. <5.0%) 33, our findings in NASH subjects were considerably lower (4.9 vs. 15–24%) than previously reported 11,34. This might be attributed to sampling DNL in the fasting state only, oral administration of deuterated water (vs. intravenous deuterated tripalmitate 11) and/or the shorter duration of stable isotope labelling compared with previous reports (14 vs. >96 h 11). Due to the nature of the stable isotopes incorporated as part of the clamp and the high rates of labelled glucose infusions required to maintain fasting glycaemia, we were unable to assess the rates of DNL associated with hyperinsulinaemia. It is important to note, however, that Donnelly et al. previously reported that the majority of lipid accumulation in NASH was attributed to adipose-derived NEFA (59%), rather than DNL (26%) 11.

We demonstrated severe adipose tissue dysfunction in patients with NASH using a variety of assessments including adipose IR index, INS-½-max NEFA, adipose tissue microdialysis and circulating adipocytokines. The discrepancy between high fasting leptin and low circulating levels of adiponectin provided further evidence of abnormal adipose tissue function. Indeed, a growing body of evidence indicates that the primary defect in NASH subjects occurs in adipose tissue 14, from which triglyceride-derived toxic metabolites including the NEFA pool, impair insulin signalling in both skeletal muscle and liver tissue (‘lipotoxicity’). A vicious cycle of hepatic, muscle and adipose tissue dysfunction ensues, leading to development of a pathogenic circulating milieu of high levels of insulin, glucose, NEFA and pro-inflammatory cytokines (e.g. hsCRP, IL-6, TNF-α, and CCL-2), all of which were observed in our patients with NASH.

Traditionally, VAT has been recognized as the major contributor to hepatic IR and lipotoxicity 35, due to its close proximity to the portal vein and concentration of inflammatory mediators 16,17. However, as VAT only contributed to 15–20% of circulating NEFA pool 18,19, researchers proposed that either VAT exerted its effects via other non-NEFA factors including adipocytokines 35 or that abdominal SAT plays an important role in lipotoxicity 36. Several studies have linked abdominal SAT with IR using euglycaemic clamps in subjects with and without metabolic syndrome 20–23, but our data is one of the first to report depot-specific dysfunction in biopsy-proven NASH subjects. Previous studies in NASH patients have solely relied on circulating NEFA to provide estimates of adipose IR 8,9,12,13,32, which are more reflective of whole-body lipolysis, rather that depot-specific 37. By directly measuring interstitial fluid concentrations of glycerol, we report novel insights into the degree of abdominal SAT IR and lipolysis in patients with NASH. The greater magnitude of resistance to the anti-lipolytic effect of insulin in SAT (sixfold vs. controls) in comparison to whole-body adipose (threefold vs. controls) in our study may well reflect depot-specific IR, in which abdominal SAT is the major source of lipotoxicity in NASH. Interestingly, using paired adipose and liver biopsies from patients undergoing bariatric surgery Tordjman et al. have recently shown that deep SAT (and not superficial SAT) has an inflammatory profile (i.e. IL-6 gene, macrophage accumulation) similar to VAT in NASH subjects 38.

One hypothesis is that abdominal SAT acts as ‘buffer’ for excess calorific intake and triglyceride deposition. When SAT fails to match the demand, as might be the case in NASH subjects, adipose hypertrophy, inflammation (via macrophage recruitment via CCL-2) and local IR sequentially develop. The resultant localized excess NEFA, as reported here, can result in an overspill of triglyceride-derived toxic metabolites into VAT and subsequently the liver 39.

The role of ethnicity in adipose IR and NASH has recently been investigated, in which Lomonaco et al. demonstrated no difference in levels of IR (EGP, fasting NEFA) between Hispanic and Caucasian cohorts with NASH, well-matched for adiposity 32. With the exception of two Italian studies 7,12, very little data exists in well-characterized patients with NASH of western European descent. Adipose IR index in our UK cohort (9.3 mmol/l µU/l) was, however, similar to that previously reported in NASH patients from southern Europe and the USA (8.0–11.9 mmol/l µU/l), all of which were 3–6.6 times higher than their respective healthy controls 8,12,13.

Our study does have limitations, in particular, the metabolic phenotype mismatch between the NASH and ‘healthy’ controls. This remains a critical challenge in real-world research, due to the high prevalence of obesity and metabolic syndrome in patients with NASH at the time of first presentation. Even though differences in adipose IR remained significant after exclusion of patients with diabetes (Figures S2, S3), we were unable to extrapolate whether our findings were independent of age and measures of adiposity. Gastaldelli et al. have reported that whole-body lipolysis (using adipose-IR index) in NASH is independent of obesity status (defined by BMI) 13, but this requires validation with robust measures of VAT and abdominal SAT mass/volume. In this study, we were unable to directly compare SAT and VAT, as real-time assessment of VAT function is not feasible in human studies. The demonstration that SAT is the dominant source of products of triglyceride hydrolysis in healthy humans 19 and our finding of marked SAT IR may have significant clinico-pathological implications in patients with NASH. This suggests that it is not simply VAT accumulation that is important in driving the pathological process. Lastly, the cross-sectional design of our study did not allow a causal relationship to be determined between dysfunctional SAT and progressive liver disease. Previous longitudinal studies (14 weeks) have shown that diet-induced decreases in MRI-measured VAT correlate with improvements in hepatic lipid content and markers of IR. However, no longitudinal studies have investigated functional changes in VAT or SAT with disease progression or regression after therapeutic intervention 40.

In summary, our study highlights that patients with NASH have marked adipose tissue dysfunction, alongside increased hepatic and muscle IR. In particular, we have drawn attention to the profound levels of IR and lipolysis in abdominal SAT, which appears disproportionate to whole-body adipose. Dysfunctional abdominal SAT likely plays a key role in NASH lipotoxicity, rather than being just a bystander to VAT. Whether this is indeed a tissue-mass effect remains to be investigated. Future prospective studies that are sufficiently powered to enable adjustment of metabolic confounders are now required to investigate the relative contribution of SAT (vs. VAT) in disease progression and the impact of novel interventions in NASH.

## Supporting Information

Figure S1 Schematic of the study design. All participants underwent a two-step hyperinsulinaemic euglycaemic clamp with stable isotope tracers (13C-glucose, deuterated water) and adipose microdialysis to determine tissue-specific insulin resistance.

Figure S2 Subjects with non-alcoholic steatohepatitis (NASH) with and without type 2 diabetes had significant abdominal subcutaneous adipose tissue (SAT) insulin resistance (IR) compared with controls. (A) SAT interstitial fluid concentrations of glycerol during the two-step hyperinsulinaemic euglycaemic clamp. (B) To determine the rate of lipolysis in SAT under basal and hyperinsulinaemic conditions area under the curve (AUC) analysis was performed using the trapezoidal method for interstitial glycerol release. Broken line/white bar = controls, solid grey line/grey bar = NASH without type 2 diabetes, sold black line/black bar = NASH with type 2 diabetes. ^&&&&^p < 0.0001 versus controls; ^+^p < 0.05, ^+++^p < 0.001, ^++++^p < 0.0001 versus basal phase. NS, non-significant.

Figure S3 Non-diabetic subjects with non-alcoholic steatohepatitis (NASH) have significantly lower levels of fasting adiponectin (A) and higher levels of fasting pro-inflammatory adipocytkines [(C) high sensitivity C-reactive protein (hs-CRP), (D) tumour necrosis factor alpha (TNF-α) and (E) interleukin-6 (IL-6)]. Higher levels of (B) leptin and (F) chemokine ligand-2 (CCL-2)/monocyte chemoattractant protein-1 (MCP-1) were seen in non-diabetic subjects with NASH, albeit not achieving significance. ^&^p < 0.05, ^&&&&^p < 0.0001 versus controls.
