# Supplementary material for: Abdominal subcutaneous adipose tissue insulin resistance and lipolysis in patients with non-alcoholic steatohepatitis
Source: Diabetes Obes Metab. 2014 Mar 11;16(7):651–60. doi: 10.1111/dom.12272 (PMC4190688; doi:10.1111/dom.12272)
Supplement: Figure S1 — Schematic of the study design. All participants underwent a two-step hyperinsulinaemic euglycaemic clamp with stable isotope tracers (13C-glucose, deuterated water) and adipose microdialysis to determine tissue-specific insulin resistance. [file dom0016-0651-sd1.pptx]

## Slide 1
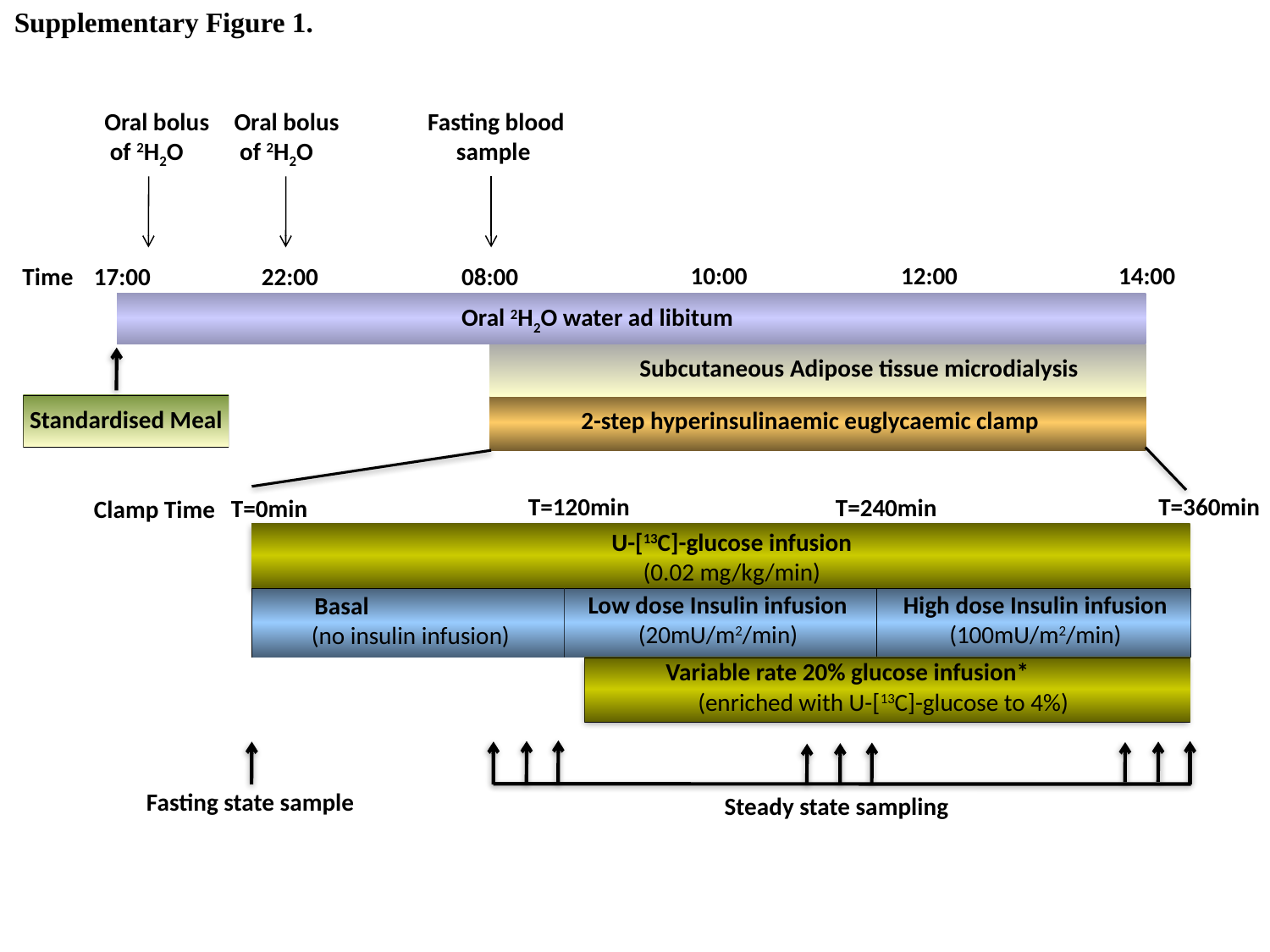

Supplementary Figure 1.
Oral bolus
 of 2H2O
Oral bolus
 of 2H2O
 Fasting blood
sample
14:00
12:00
10:00
Time
17:00
22:00
08:00
Oral 2H2O water ad libitum
Subcutaneous Adipose tissue microdialysis
Standardised Meal
2-step hyperinsulinaemic euglycaemic clamp
T=360min
T=120min
T=240min
T=0min
Clamp Time
U-[13C]-glucose infusion (0.02 mg/kg/min)
Low dose Insulin infusion (20mU/m2/min)
High dose Insulin infusion (100mU/m2/min)
Basal (no insulin infusion)
Variable rate 20% glucose infusion* (enriched with U-[13C]-glucose to 4%)
Fasting state sample
Steady state sampling
