# Supplementary figures and images for: Abdominal subcutaneous adipose tissue insulin resistance and lipolysis in patients with non-alcoholic steatohepatitis
Source: Diabetes Obes Metab. 2014 Mar 11;16(7):651–60. doi: 10.1111/dom.12272 (PMC4190688; doi:10.1111/dom.12272)

## Slide 1
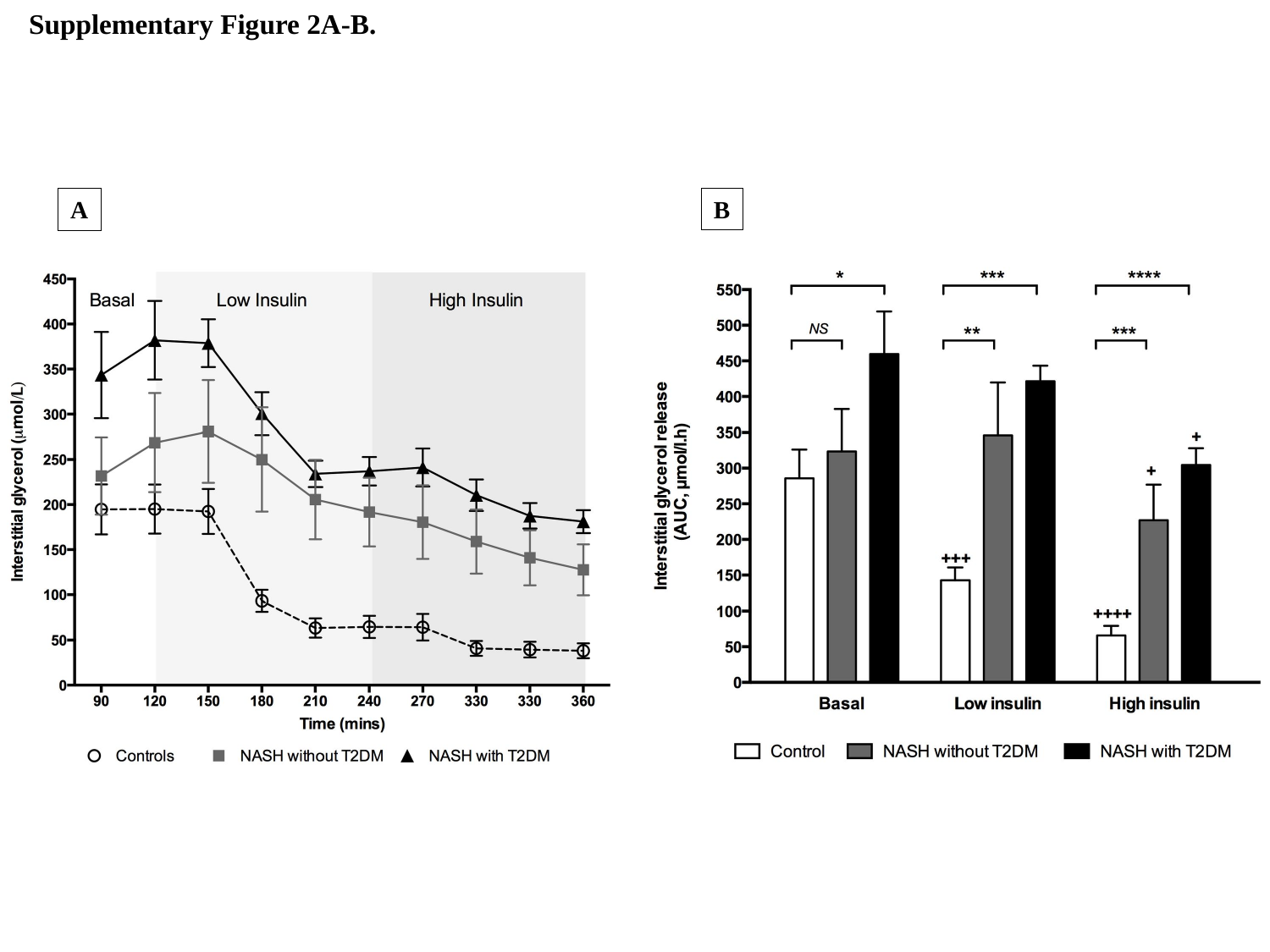

Supplementary Figure 2A-B.
B
A

Supplement: Figure S2 — Subjects with non-alcoholic steatohepatitis (NASH) with and without type 2 diabetes had significant abdominal subcutaneous adipose tissue (SAT) insulin resistance (IR) compared with controls. (A) SAT interstitial fluid concentrations of glycerol during the two-step hyperinsulinaemic euglycaemic clamp. (B) To determine the rate of lipolysis in SAT under basal and hyperinsulinaemic conditions area under the curve (AUC) analysis was performed using the trapezoidal method for interstitial glycerol release. Broken line/white bar = controls, solid grey line/grey bar = NASH without type 2 diabetes, sold black line/black bar = NASH with type 2 diabetes. &&&&p < 0.0001 versus controls; +p < 0.05, +++p < 0.001, ++++p < 0.0001 versus basal phase. NS, non-significant. [file dom0016-0651-sd2.pptx]

## Slide 1
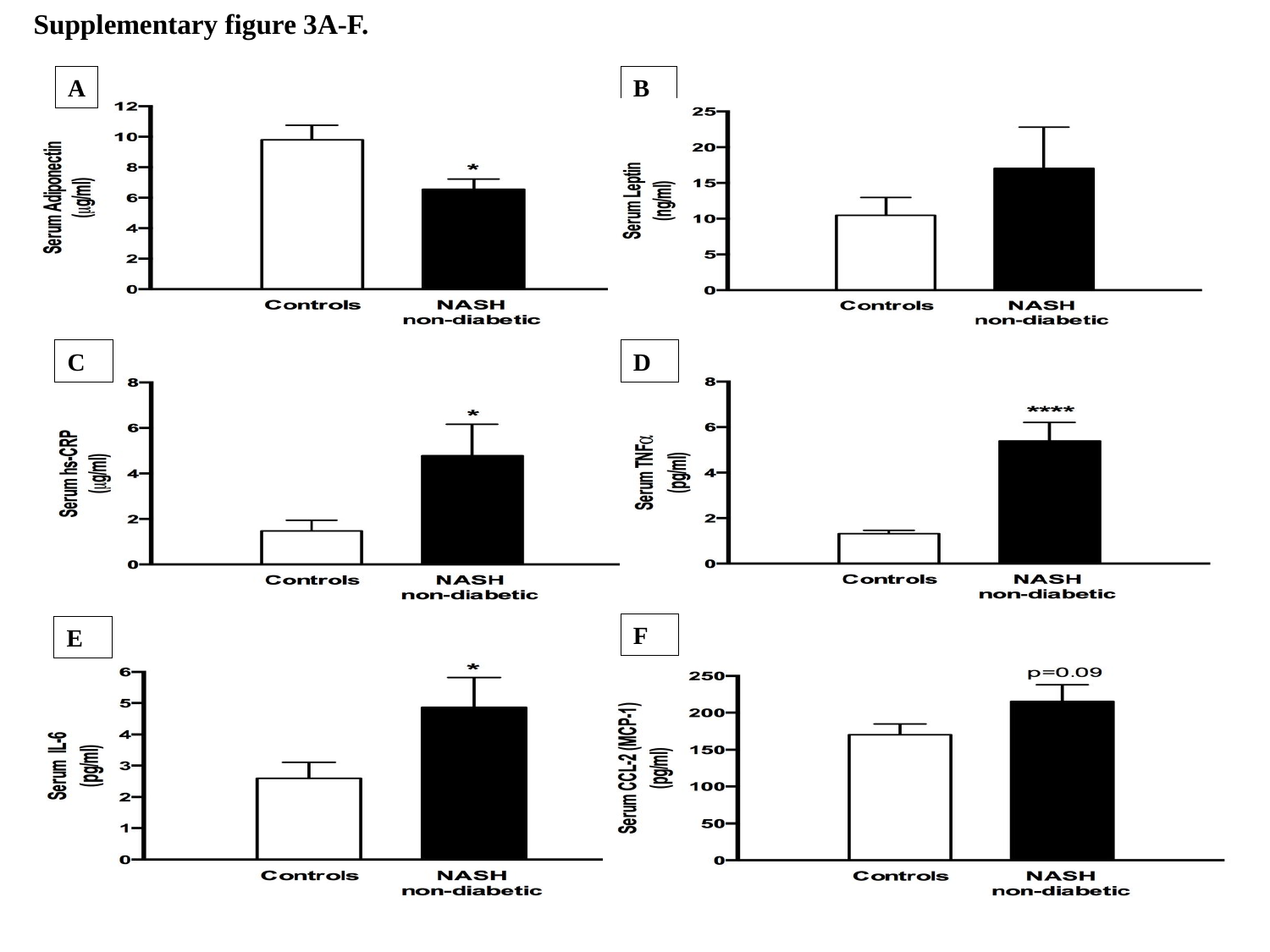

Supplementary figure 3A-F.
A
B
C
D
F
E

Supplement: Figure S3 — Non-diabetic subjects with non-alcoholic steatohepatitis (NASH) have significantly lower levels of fasting adiponectin (A) and higher levels of fasting pro-inflammatory adipocytkines [(C) high sensitivity C-reactive protein (hs-CRP), (D) tumour necrosis factor alpha (TNF-α) and (E) interleukin-6 (IL-6)]. Higher levels of (B) leptin and (F) chemokine ligand-2 (CCL-2)/monocyte chemoattractant protein-1 (MCP-1) were seen in non-diabetic subjects with NASH, albeit not achieving significance. &p < 0.05, &&&&p < 0.0001 versus controls. [file dom0016-0651-sd3.pptx]
