# Supplementary material for: Abdominal subcutaneous adipose tissue insulin resistance and lipolysis in patients with non-alcoholic steatohepatitis
Source: Diabetes Obes Metab. 2014 Mar 11;16(7):651–60. doi: 10.1111/dom.12272 (PMC4190688; doi:10.1111/dom.12272)
Supplement: Appendix S1 — Supplementary methods. [file dom0016-0651-sd4.docx]

**Supplementary material - Armstrong MJ et al**

**Supplementary Methods:**

*Clinical and biochemical parameters:*

Type 2 diabetes was defined by past medical history, 75g 2-hour oral glucose tolerance test, glycated haemoglobin (HbA1c) and/or fasting glucose (WHO 2011). Measurements of weight (kg), height, systolic/diastolic blood pressure and total body/truncal fat mass (bioimpedance) were recorded. Body mass index (BMI) was defined as weight in kilograms divided by the square of the height in metres (kg/m^2^). Fasting blood samples (0800 hours) were analysed for full blood count, urea, creatinine and electrolytes, thyroid stimulating hormone (TSH), lipid profile, liver function tests (LFT), HbA1c and plasma glucose using standard laboratory methods (Roche Modular system, Roche Ltd, Lewes, UK).

Serum Insulin was measured using a commercially available colorimetric ELISA (Mercodia, Uppsala, Sweden), with an in-house coefficient of variation of <5%. Serum non-esterified fatty acids (NEFA) were measured in-house using a colorimetric commercial assay (Zen-Bio, Research Triangle Park, NC, USA), with a coefficient of variation between 8.0-9.1%. Both were performed according to the manufactures’ instructions. Serum caspase-cleaved cytokeratin-18 (CK-18 M30) and the Enhanced Liver Fibrosis (ELF) Test were performed at study entry to assess hepatic apoptosis and fibrosis, respectively. Serum CK-18 M30 was measured in accordance with the manufacturers’ guidance using a commercially available colorimetric ELISA (M30 Apoptosense ELISA Kit; PEVIVA AB, Bromma, Sweden), with an in-house coefficient of variation of <5%. The ELF test, which combines three direct serum markers of fibrosis (hyaluronic acid (HA), procollagen III amino terminal peptide (PIIINP) and tissue inhibitor of metalloproteinase 1 (TIMP-1)) using an algorithm developed by the European Liver Fibrosis Group (Rosenberg W, 2004) was performed on fasting serum stored at -80 degrees by a commercial laboratory (iQUR Ltd, Royal Free Hospital, London, UK).

*Circulating adipocytokines and inflammatory markers:*

Fasting serum levels of Adiponectin, Leptin, Resistin, TNF-α, hs-CRP, IL-6, IL-17, CCL-2 (MCP-1, Monocyte Chemotactic Protein-1), CCL-3 (MIP-1α, Macrophage Inflammatory Protein 1α), CCL-4 (MIP-1β, Macrophage Inflammatory Protein 1β) and CCL-5 (RANTES, Regulated on Activation, Normal T cell Expressed and Secreted) were quantified using the commercially available multiplex bead immunoassays (Fluorokine® Multi-Analyte Profiling; R&D Systems, Abingdon, United Kingdom) for the Luminex™ 200 Platform (Luminex Corporation, The Netherlands). Six-point standard curves for each cytokine on the Human Obesity Base Kit (cat. No. LOB000) and Human Base Kit A (cat. No. LOB000) were generated by reconstitution of the Standard Cocktail with Calibrator Diluent RD6-46, as per manufacturers instructions. The Biorad Bioplex-Manager (version 6.0) software was used on the Luminex ™ 200 machine for acquisition and analysis. The in-house minimum detection limits (coefficient of variations, CV %) were as follows: 438.5 pg/ml for Adiponectin (CV 2.1-9.5%); 58.7 pg/ml for Leptin (CV 2.0-8.1%); 46.4 pg/ml for Resistin (CV 0.9-5.2%); 1.19 pg/ml for TNF-α (CV 1.1-6.6%); 36.0 pg/ml for hs-CRP (CV 0.0-9.0%); 0.46 pg/ml for IL-6 (CV 1.8-6.7%); 0.45 pg/ml for IL-17 (CV 0.44-20.9%); 3.75 pg/ml for CCL-2 (CV 0.59-9.4%); 18.5 pg/ml for CCL-3 (CV 0.0-12.8%); 8.10 pg/ml for CCL-4 (CV 0.77-13.8%); and 2.96 pg/ml for CCL-5 (CV 0.71-18.7%).

*Stable Isotope Mass Spectrometry analysis:*

The enrichment of U-[^13^C]-glucose in plasma was determined by gas chromatography-mass spectrometry (model 5973; Agilent technologies, Cheshire, UK). Deuterium enrichment of the body water pool was measured using the Gasbench II (<http://vsites.unb.br/ig/labo/isotopos/productPDF_27060.pdf>), an automated H_2_/H_2_O equilibration device, coupled on line to a ThermoFinnigan Deltaplus XP Isotope Ratio Mass Spectrometer (IRMS; ThermoFinnigan MAT GmbH, Bremen, Germany). In brief, after adding 200µl of plasma sample and inserting platinum catalyst to a borosilicate sample vial, the vial is capped and automatically flushed with 2% H_2_ in He equilibration gas. After an equilibration time of 40 minutes, the ^2^H/^1^H enrichment of the head space gas is sampled and analysed automatically (mean of 10-fold measurement) on the IRMS using 2% H_2_ in He as reference gas. The in house coefficient of variation of this assay is <2% for naturally enriched samples and <0.5% for samples with a ^2^H/^1^H ratio 0.001 > natural background.

Deuterium enrichment in the palmitate fraction of total plasma triglycerides was measured on an automated GC/TC/IRMS system (ThermoFinnigan Delta Pus XP; <http://www.thermo.com/eThermo/CMA/PDFs/Product/productPDF_27059.pdf>). In brief, the lipid fraction was extracted from 1.5 ml of plasma as described by Folch et al (Folch J, 1957) and the TG fraction isolated by solid phase extraction (Bond Elut NH2-Aminopropyl columns). After transmethylation of the TG fraction (Lepage J, 1987) the ^2^H/^1^H ratio in palmitate methylester was measured via a GC separation of the methylated fatty acids followed by pyrolytic conversion of the palmitate methylester into CO and H_2_, followed by online continuous flow measurement of the ^2^H/^1^H ratio in the separated H_2_ peak by the ThermoFinningan Deltaplus XP IRMS. The in house coefficient of variation of this assay is <5% over the sample range observed in this study (^2^H/^1^H ratio 0.00000-0.0004 > natural background). The ^2^H/^1^H ratio of both the body water pool and of the palmitate fraction of total plasma TG were corrected against enrichment curves.

**Additional references:**

Rosenberg WMC, Voelker M, Thiel R, et al. Serum markers detect the presence of liver fibrosis: a cohort study. Gastroenterology 2004;127:1704-1713.

Folch J, Lees M, Sloane Stanley GH. A simple method for the isolation and purification of total lipides from animal tissues. J Biol Chem 1957;226:497-509.

Lepage G, Roy CC. Direct transesterification of all classes of lipids in a one-step reaction. J Lipid Res 1986;27:114-120.
